# Dr. W. W. Sheffield

**Published:** 1897-12

**Authors:** 


### Dr. W. W. Sheffield.

Dr. Sheffield died at his home in New London, Conn., November —, 1897. Dr. Sheffield was one of the older practitioners in Connecticut, having been in practice in that city about thirty-five years. He came to New London in 1852 and began his career as a dentist in the office of Dr. J. A. G. Comstock. The degree of Doctor of Dental Surgery was conferred upon him (honorary), by the Ohio College of Dental Surgery. He was a very successful practitioner and was engaged in full practice up till about two years ago when he was stricken with paralysis, from which he only partially recovered, not sufficiently however to resume full practice. A recent attack was so severe as to completely break him down, and within a brief time he died. He leaves a wife, who was Miss Harriet P. Brown, and a son, Dr. L. T. Sheffield, a practitioner in New York City. Dr. Sheffield while in the high tide of his professional practice, was a leading man in his State. He was at one time a prominent member of the American Dental Association, and was elected its Vice-President. Some years ago misfortune overtook him and undoubtedly did much to break down his vigorous constitution and prepare the way for the last and fatal attack. Dr. Sheffield possessed a warm and genial nature, and was always true to his friends, of whom he had a host. The widow and son will have the sincere sympathy of all his friends in this their great bereavement.

